# CApecitabine plus Radium-223 (Xofigo™) in breast cancer patients with BONe metastases (CARBON): study protocol for a phase IB/IIA randomised controlled trial

**DOI:** 10.1186/s13063-019-3643-6

**Published:** 2020-01-15

**Authors:** Rob Coleman, Janet Brown, Emma Rathbone, Louise Flanagan, Amber Reid, Jessica Kendall, Sacha Howell, Chris Twelves, Carlo Palmieri, Anjana Anand, Iain MacPherson, Sarah Brown

**Affiliations:** 1 The University of Sheffield, Sheffield, UK; 2 Calderdale and Huddersfield NHS Foundation Trust, Huddersfield, UK; 3 Clinical Trials Research Unit, Leeds Institute of Clinical Trials Research, University of Leeds, Leeds, UK; 4 The Christie NHS Foundation Trust, Manchester, UK; 5 St James’s University Hospital, Leeds, UK; 6 Leeds Institute of Cancer Studies and Pathology, University of Leeds, Leeds, UK; 7 Clatterbridge Cancer Centre NHS Foundation Trust, Liverpool, UK; 8 University of Liverpool, Liverpool, UK; 9 Nottingham University Hospitals NHS Trust, Nottingham, UK; 10 Institute of Cancer Sciences, University of Glasgow, Glasgow, UK

**Keywords:** Radium-223, Capecitabine, Bone metastases, Bone turnover markers, Breast cancer

## Abstract

**Background:**

A substantial proportion of breast cancer patients develop metastatic disease, with over 450,000 deaths globally per year. Bone is the most common first site of metastatic disease accounting for 40% of all first recurrence and 70% of patients with advanced disease develop skeletal involvement.

Treatment of bone metastases currently focusses on symptom relief and prevention and treatment of skeletal complications. However, there remains a need for further treatment options for patients with bone metastases. Combining systemic therapy with a bone-targeted agent, such as radium-223, may provide an effective treatment with minimal additional side effects.

**Methods/design:**

CARBON is a UK-based, open-label, multi-centre study which comprises an initial safety phase to establish the feasibility and safety of combining radium-223 given on a 6-weekly schedule in combination with orally administered capecitabine followed by a randomised extension phase to further characterise the safety profile and provide preliminary estimation of efficacy.

**Discussion:**

The CARBON study is important as the results will be the first to assess radium-223 with chemotherapy in advanced breast cancer. If the results find acceptable rates of toxicity with a decrease in bone turnover markers, further work will be necessary in a phase II/III setting to assess the efficacy and clinical benefit.

**Trial registration:**

ISRCTN, ISRCTN92755158, Registered on 17 February 2016.

**Electronic supplementary material:**

The online version of this article (10.1186/s13063-019-3643-6) contains supplementary material, which is available to authorized users.

## Background

### Metastatic breast cancer

Despite significant advances and improvements in outcomes following breast cancer, a significant proportion of patients still develop metastatic disease with bone being the most common first site for distant metastasis. Metastatic tumour development is thought to follow complex interactions between the tumour cell and the bone microenvironment allowing occupation of the haematopoietic stem cell and other cellular niches in the bone marrow by tumour cells. Tumour-derived factors attract and stimulate osteoclasts, increasing bone turnover and releasing bone-activated growth factors and cytokines [1]

The skeletal lesions seen in association with breast cancer are most commonly osteolytic and associated with significant morbidity due to the skeletal complications, termed skeletal-related events (SREs): severe bone pain requiring radiation, pathological fracture, spinal cord or nerve root compression, hypercalcaemia and the requirement of surgery or radiation to bone. However, there is usually an osteoblastic component that is manifested by the visualisation of bone metastases on radionuclide bone scans and elevation of osteoblastic bone markers such as bone-specific alkaline phosphatase (B-ALP). Although sometimes identified on imaging tests, most SREs are associated with symptoms when they are known as symptomatic skeletal events (SSEs). The median survival time after the development of bone metastases is approximately 2–3 years.

Bone turnover markers, biological indicators of either bone resorption or bone formation, are associated with clinically relevant endpoints. *N*-telopeptide of type-I collagen (NTX) is derived from the breakdown of type-1 collagen during bone remodelling and reflects the rate of bone resorption. Elevated levels of urinary NTX (uNTX) are associated with significantly increased rate of SREs and disease progression amongst a solid tumour population, in addition to increased mortality. B-ALP is a bone-formation marker which is similarly associated with negative outcomes in patients with elevated levels [2].

There are many treatment options available to patients with advanced breast cancer including surgery, radiotherapy and systemic therapies. Current treatment of bone metastases also focusses on symptom relief and treatment and prevention of SREs. Anti-resorptive agents, such as bisphosphonates or denosumab, delay SREs and are subsequently now widely established as standard therapy for such patients [3–6]. Additionally, bisphosphonates suppress bone turnover markers, with their normalisation or degree of suppression correlating with reduced SREs and death rate [7]. However, there remains a need for further treatment options for patients with bone metastases to improve survival more than 2–3 years. Combining systemic therapy with a bone-targeted agent, such as radium-223, may provide an effective treatment with minimal additional side effects.

### Radium-223

Radium-223 dichloride (radium-223) is a novel alpha-emitting pharmaceutical that has been developed for the treatment of bone metastases. The product is based on the alpha-emitting radionuclide radium-223. The intrinsic bone-targeting property of radium-223 compounds is similar to that of other alkaline earth elements, like calcium. The characteristics of alpha-emitting radionuclides have benefits over beta-emitting radionuclides for bone targeting. Firstly, radium-223 emits alpha-particles with high linear-energy transfer and a radiation range limited to less than 100 μm [8–11]. This generates a highly localised and effective radiation zone with high probability of inducing double-strand DNA breaks in the cancer cells. LOn the other hand, beta-emitting radiopharmaceuticals, such as strontium, emit particles with lower energy, mainly inducing single-strand breaks which are more easily repaired. Additionally, beta-particles typically have 30–80 times longer radiation range compared to alpha-particles. An alpha-emitting radiation source located in a target tissue, such as bone metastases, will deliver higher energy radiation to a more localised volume than beta emitters, thereby reducing exposure of surrounding normal tissues and leading to less toxicity, importantly less myelotoxicity. Radium-223 received Food and Drug Administration (FDA) approval in 2013 for the treatment of patients with castration-resistant prostate cancer (CRPC) and bone metastases [12].

### Pre-clinical data

Pre-clinical data in an experimental skeletal metastasis model in nude rats inoculated intravenously with human breast cancer cells demonstrated a significant radium-223 anti-tumour effect and showed significantly symptom-free survival [13].

### Clinical data

Since radium-223 began clinical development in 2001 there have been several phase I studies in patients with bone metastases from prostate or breast cancer. These showed that the drug is quickly eliminated from blood, taken up by bone and excreted via the small intestine [13]. The kidneys, bladder and urethra are, therefore, exposed to minimal amounts of radiation. No specific uptakes were seen in the heart, liver, kidney, gallbladder, stomach or spleen. The highest absorbed doses were measured in the bone and red marrow.

Pain relief was observed in studies using both single doses and multiple doses of radium-223, with better pain relief being observed with the higher doses [14, 15]. Decreases in B-ALP were observed at all dose levels, similarly more pronounced at the higher doses [14].

Multiple dosing at 6-weekly intervals was evaluated in the BC1–04 study, a phase II study in patients with symptomatic or non-symptomatic hormone-refractory prostate cancer. One hundred and twenty-two patients received three doses of 25, 50 or 80 kBq/kg. A significant dose-response relationship was seen, with an increasing proportion of prostate-specific antigen (PSA) responders and B-ALP responders with increasing dose. The two highest dose levels had a greater effect than the lowest dose levels, with no significant difference between 50 and 80 kBq/kg [16].

### Prostate cancer – single agent and combination with chemotherapy

The majority of studies to date have been in prostate cancer using radium-223 as a single agent. In a placebo-controlled, phase II study in hormone-refractory prostate cancer patients with bone metastases, patients were assigned 4 × 50-kBq/kg radium-223 injections (*n* = 33) or saline injections (*n* = 31) given every 4 weeks [17]. The median relative change in B-ALP was significantly greater in the treatment arm compared with the control arm (− 65.6% vs. 9.3%; *P* < 0.0001). Toxicity from radium-223 is mild and tolerable. The most common Adverse Events (AEs) were reversible myelosuppression, diarrhoea, nausea and vomiting, with all reported as mild or moderate in severity. A combined analysis of 300 patients treated with radium-223 showed that radium-223 is well tolerated with a low propensity for haematological toxicity [18].

The phase III registration study of radium-223 (ALSYMPCA) was performed in 921 patients with CRPC and bone metastases and compared best supportive care with radium 50 kBq/kg every 4 weeks for six cycles or placebo. The primary endpoint was overall survival. Patients who received radium-223 had a significant 3.6-month improvement in median survival (hazard ratio (HR) = 0.70; 95% confidence interval (CI), 0.58–0.83; *P* = 0.001) compared with patients who received placebo [12]. These benefits were achieved without significant toxicity and with additional benefits on the frequency of SSEs, even in the presence of concomitant bisphosphonates [19].

### Rationale for using radium-223 to treat metastatic breast cancer

The mechanism of action and efficacy of radium-223 in prostate cancer suggests that the agent may be effective in other cancers that have a propensity to metastasise to the bone. This was confirmed in an open-label, phase IIa, non-randomised study of radium-223 in breast cancer patients with bone-dominant disease. This study evaluated any clinically relevant effect on bone markers in breast cancer patients who had progressed on endocrine treatment and were no longer considered suitable for further endocrine therapy.

Radium-223 significantly reduced uNTX and B-ALP from baseline to end of treatment. Radium-223 was safe and well tolerated [16]. Therefore, it is important to assess the toxicity and efficacy of radium-223 in a population of patients that commonly requires chemotherapy. Currently, larger randomised trials are evaluating the addition of radium-223 to an aromatase inhibitor alone or with everolimus (ClinicalTrials.gov Identifiers: NCT02258451 and NCT02258464). The results are anticipated in 2020. Combinations with chemotherapy are also of interest for treatment later in the clinical course. Capecitabine was selected due to its frequent use as a single agent in breast cancer and relative lack of myelotoxicity. The combination may, therefore, target both bone metastases and visceral metastases.

There are ongoing studies combining radium-223 with endocrine treatments (NCT02258464 and NCT02258451) and a small safety study (n=15) has evaluated the combination of paclitaxel and radium-223 in a mixed population of cancer patients [1], but thisThis will be the first study to specifically assess radium with chemotherapy in advanced breast cancer.

## Methods/design

### Study aims and objectives

The purpose of this study is to determine the safety profile and clinical relevance of the combination of radium-223 and capecitabine in breast cancer patients with bone metastases. The study protocol and this manuscript have been written in accordance with Standard Protocol Items: Recommendations for Interventional Trials (SPIRIT) guidelines.

#### Primary objectives

- To evaluate the safety and toxicity of the combination of radium-223 and capecitabine
- To preliminarily estimate if multiple intravenous (i.v.) injections of radium-223 plus capecitabine will have any clinically relevant effect on uNTX in breast cancer patients with bone metastases, with or without other sites of disease

The sample size calculation is based solely on the primary toxicity endpoint. Primary analysis of the uNTX endpoint is focussed on estimation only, i.e. no formal hypothesis testing will be carried out for the purpose of decision making. No formal comparisons will be made.

#### Secondary objectives

To evaluate the effect of radium-223 on other bone turnover markers (*N*-terminal propeptide of procollagen type-1 (P1NP), serum C-telopeptide (CTX), pyridinoline cross-linked carboxyterminal telopeptide (1CTP), B-ALP)

- To evaluate pain scores and quality of life
- To evaluate time to first SSE – defined as any of: use of external-beam radiotherapy to relieve skeletal symptoms; new symptomatic pathological vertebral or non-vertebral bone fracture; spinal cord compression; tumour-related orthopaedic surgical intervention
- To evaluate time to progression of bone disease and time to progression of extraskeletal disease

### Study design

The trial is designed as a randomised, controlled, open-label, multi-centre, phase IB/IIA study. It comprises an initial safety phase to establish the feasibility and safety of combining radium-223 at the recommended dose of 55 kBq/kg given on a 6-weekly schedule to enable combination with orally administered capecitabine administered with the standard of care schedule (2 weeks of capecitabine followed by 1 week off treatment). Recruitment to the initial safety phase utilises a 3 + 3 design. If the treatment in the initial safety phase proves to be feasible and safe, the randomised extension phase will open to recruitment. The extension phase of the study aims to characterise the safety profile and provide preliminary estimation of efficacy.

**Fig. 1:**
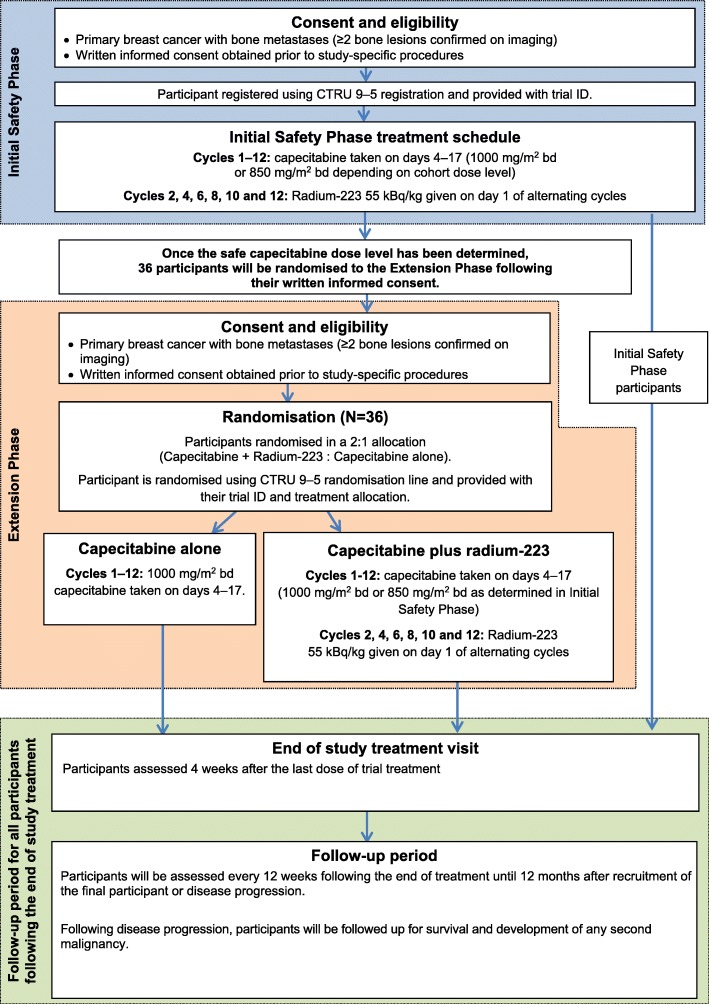
Participant pathway

**Fig. 2:**
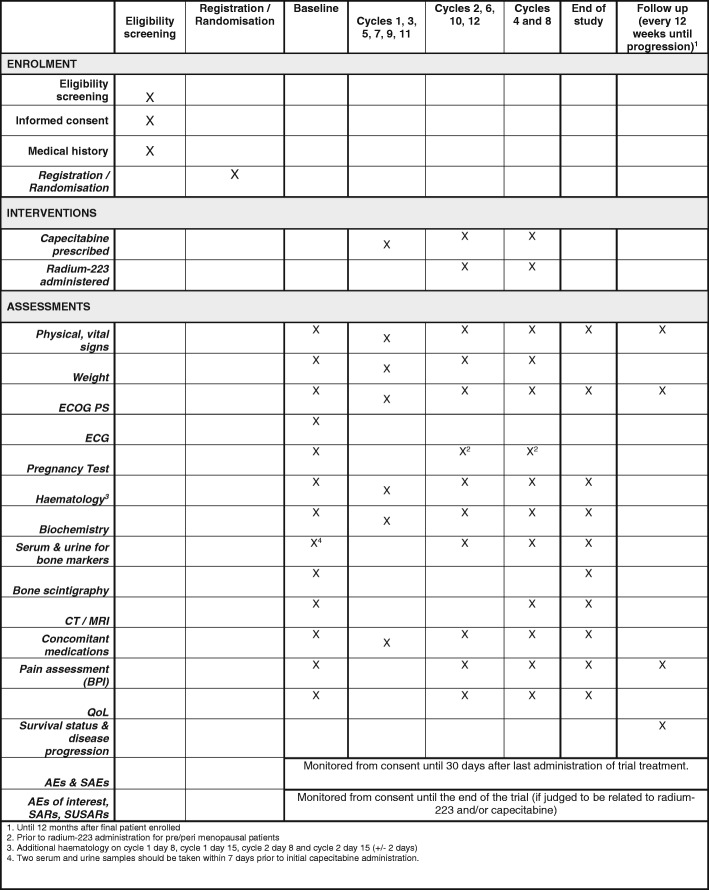
Standard Protocol Items: Recommendations for Interventional Trials (SPIRIT) Figure. Schedule of enrolment, interventions and assessments for participants who are suitable for enrolment to the trial

The participant pathway can be seen schematically in Fig. 1 and a populated Standard Protocol Items: Recommendations Figure is also provided in Fig. 2.

### Study population

Patients are eligible if they have histological evidence of primary breast cancer with imaging evidence of bone metastases (with or without soft tissue, lymph node or visceral metastases; brain metastases allowed if stable and untreated for ≥ 8 weeks) and systemic chemotherapy with capecitabine is felt to be appropriate by the treating physician. They must have received no more than two lines of chemotherapy in the metastatic setting and prior cytotoxic therapy must have been completed 28 days or more prior to initiation of study treatment. They must have an Eastern Co-operative Oncology Group (ECOG) performance status 0–2 and a life expectancy of 6 or more months. Patients are required to have appropriate haematological and biochemical parameters, to be aged 18 years or over and be able and willing to consent and comply with study treatment, visits and required investigations.

Patients are excluded from the study if they are pregnant or breast feeding; received an investigational drug within 4 weeks prior to the first study treatment; received external-beam radiotherapy within 4 weeks prior to the first study treatment; they have the presence of imminent or established spinal cord compression based on clinical findings and/or magnetic resonance imaging (MRI); presence of other currently active (diagnosis within the last 5 years) malignancy (except treated non-melanoma skin cancer (basal or squamous), carcinoma in situ of the cervix and superficial bladder cancers). Patients are also excluded if they have had severe and unexpected reactions to fluoropyrimidine therapy or have been diagnosed with dihydropyrimidine dehydrogenase deficiency; received a blood transfusion or erythropoietin within 4 weeks of study treatment; are hypersensitive to capecitabine or any of its excipients; have received treatment with sorivudine or its chemically related analogues, such as brivudine; and are receiving treatment with phenytoin or warfarin. Patients with any other serious illness or medical condition will be excluded, such as, but not limited to: any uncontrolled infection, clinical heart failure (NYHA Heart Failure Class III or IV), active Crohn’s disease or ulcerative colitis, bone marrow myelodysplasia, uncontrolled coronary artery disease, active peptic ulcers, malabsorption.

### Sample size

Approximately 48 participants will be recruited across both phases of the trial.

#### Initial safety phase

**Fig. 3:**
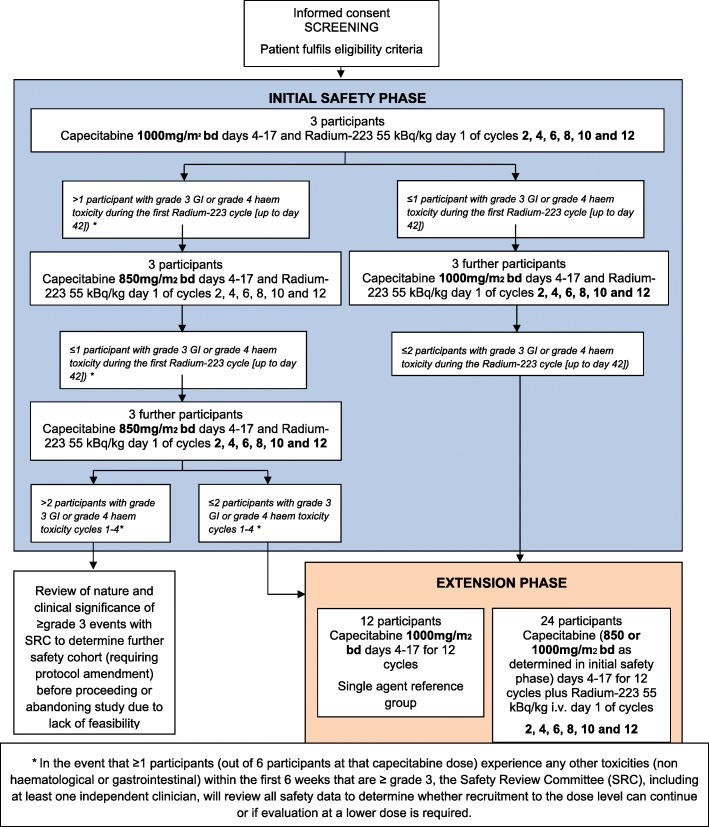
Dose Limiting Toxicity (DLT) schema

A minimum of six and a maximum of 12 evaluable participants will be recruited into the initial safety phase. This is based on the number of participants experiencing Dose Limiting Toxicities (DLTs), using a modified 3 + 3 approach (see Fig. 3).

#### Extension phase

An additional 36 participants will be recruited to the extension phase, and will be randomised on a 1:2 ratio to receive either:

- Orally administered capecitabine alone 1000 mg/m^2^ twice a day (bd) or, capecitabine 850 or 1000 mg/m^2^ bd (as determined in the initial safety phase) plus radium-223 55 kBq/kg by i.v. injection on day 1 of cycle (2, 4, 6, 8, 10 and 12) of capecitabine for a total of six injections.

The sample size for the extension phase is based upon the primary toxicity endpoint of grade-III/IV toxicity. With 24 participants in the capecitabine plus radium-223 arm, this provides approximately 80% power to exclude a grade-III/IV diarrhoea rate of 25% from the upper limit of a one-sided 85% CI, assuming a rate of approximately 10% with capecitabine alone. If no more than 3/24 participants experience grade-III/IV diarrhoea the upper limit of the one-sided 85% CI will exclude 25%.

The control arm is included to provide concurrent standard of care data for interpretation only. No formal comparisons between arms will be made.

### Recruitment

Participants are recruited from NHS hospitals throughout the UK which are approved to participate in this study having obtained requisite ethical and management approval. There are four centres open to recruitment; Weston Park Hospital in Sheffield, St James’s Hospital in Leeds, The Christie Hospital in Manchester and the Clatterbridge Cancer Centre in Liverpool. These centres have been selected based on their proven track record in delivering early phase studies and experience with radium-223. Further centres may be considered if the recruitment target is not being met. The estimated accrual for this study is two to three participants a month. Thus, participant accrual is expected to be completed within 18 months.

### Study treatment

A maximum of 12 cycles of combination therapy is given unless disease progression or unacceptable toxicity are encountered. A cycle is 21 days in accordance with the standard administration of capecitabine.

A maximum of two doses of capecitabine are being investigated in this study; 850 mg/m^2^ bd and 1000 mg/m^2^ bd. The starting dose is 1000 mg/m^2^ bd which is in accordance with the standard administration of capecitabine. Capecitabine is delivered on days 4–17 for up to 12 cycles to provide a 3–4-day window before and after radium-223 to minimise any risk of potentiating normal tissue radiation sensitivity. After cycle 12, patients may continue with capecitabine off study as per standard of care, if they are continuing to receive clinical benefit.

Radium-223 is administered at 55 kBq/kg. This dose has been selected based on clinical data from previous studies in prostate cancer and breast cancer which have demonstrated efficacy and tolerability [14, 16–18]. Radium-223 is administered as a slow i.v. injection on day 1 of alternating cycles, starting at cycle 2 to provide one cycle of safety information in each participant with capecitabine alone.

#### Initial safety phase

The safety data for the first cohort is reviewed by the Safety Review Committee and the capecitabine dose will either be expanded to a further three participants or de-escalated to 850 mg/m^2^ bd depending on the frequency and severity of toxicity experienced.

Toxicity data for each subsequent cohort is reviewed prior to dose expansion after three participants have been recruited and observed for DLTs, according to Fig. 3.

DLTs are assessed during the second cycle of capecitabine treatment up to the administration of cycle 3 day 1 and will be defined as the following events:

- Gastrointestinal toxicity of ≥ grade III lasting > 48 h despite adequate supportive care measures
- Haematological toxicity ≥ grade IV lasting > 7 days despite adequate supportive care measures which should exclude the use of bone marrow growth factors

Gastrointestinal toxicity ≥ grade III or haematological toxicity≥ grade IV experienced by participants during the first cycle, i.e. up to the administration of cycle 2 day 1 are not classed as DLTs as they are related to the capecitabine only.

The number of participants recruited to the initial safety phase, and the dose carried forward to the randomised extension phase is determined by the number of DLTs in line with the DLT schema.

#### Evaluability

Participants can miss no more than 50% of doses in the first two treatment cycles in order to be evaluable for DLT in the initial safety phase. Participants missing more than 50% of doses for reasons other than toxicity without experiencing a DLT will be replaced.

### Assessments

Participants are assessed clinically at baseline, on day 1 of each cycle, with additional haematology assessments during cycles 1 and 2 (days 8 and 15, respectively), at the end of study visit, and then at the 12-weekly follow-up visits.

Planar bone scintigraphy/SPECT ± computerised tomography (CT) is performed at baseline and at the end of study visit, and when clinically indicated. Chest/abdominal/pelvic CT or MRI is performed at baseline, weeks 12 and 24 after the initiation of treatment, at the of study visit and when clinically indicated.

Serum and second voided urine samples are collected to analyse changes in bone turnover markers at baseline, day 1 of alternating cycles starting at cycle 2, and at the end of the study visit.

Quality of life is assessed using EORTC QLQ-C30 and QLQ-Bone Metastases Module (QLQ-BM22) which will be completed by the participant prior to the first administration of trial treatment, day 1 of alternating cycles starting at cycle 2, and at the end of study visit.

Response is evaluated using RECIST v1.1 with comparisons to baseline evaluation.

### Data collection and management

All protocol-required information is completed at sites onto paper Case Report Forms by research staff, with the exception of quality-of-life information which will be completed directly by participants. Overall trial and data management is provided by the Leeds Clinical Trials Research Unit (CTRU), including monitoring of data for quality and completeness.

The independent Safety Review Committee (SRC) meets regularly to review the safety and ethics of the study by regularly reviewing the safety data during the dose escalation and expansion phases.

### Statistical analysis

Statistical analysis is the responsibility of the CARBON CTRU trial statistician. A full statistical analysis plan will be written before any formal analyses are undertaken.

During the initial safety phase, after every cohort of three participants, the CTRU trial statistician produces a summary of DLTs and AEs at each dose level, detailed safety listings and treatment compliance data for all participants in the study. The final analysis of primary endpoints and all secondary endpoints will take place when all participants have been followed up for at least 12 months.

The analysis population for the initial safety phase includes any participant who has received at least one complete cycle of capecitabine and radium-223. For the extension phase intention-to treat (ITT) population, per-protocol and safety analysis populations will be used. Statistical analysis is summarised by treatment arm.

### Primary endpoint analysis

#### Dose Limiting Toxicities (initial safety phase)

The number of participants experiencing DLTs within the first cycle of radium-223 treatment will be presented, with descriptive summaries of the specific DLTs observed.

#### Frequency of CTC grade-III/IV toxicities with a focus on diarrhoea as the primary toxicity (extension phase)

The number of participants experiencing any grade-III/IV toxicity will be presented by arm, across all cycles as well as by each cycle. Toxicities should be graded using the National Cancer Institute Common Terminology Criteria for Adverse Events version 4.03 (NCI CTCAE).

#### Decrease in uNTX from baseline to end of cycle 5 (approximately 15 weeks post trial entry) (extension phase)

The proportion of participants categorised as responders (≥ 30% reduction in uNTX from baseline) at the end of cycle 5 will be presented by arm.

### Secondary endpoint analysis

#### Safety endpoints

- The number of Serious Adverse Events (SAEs), Serious Adverse Reactions (SARs) and Serious Unexpected Adverse Reactions (SUSARs) will be summarised descriptively by arm, causality, seriousness and body system
- The worst AE for each participant will be summarised by arm, overall and by treatment cycle. The proportion of participants experiencing each toxicity will be summarised by maximum NCI CTCAE (V4.03) grade by arm
- Dose delays and reductions will be summarised by arm overall and by treatment cycle

#### Efficacy endpoints

- Descriptive and graphical summaries of the changes in serum bone turnover markers will be presented by arm. Multi-level repeated-measures modelling will also be used to estimate differences between the two treatment arms over time
- Time to occurrence of first symptomatic skeletal event (SSE) will be calculated using the Kaplan-Meier method. Median time to first SSE will be presented with corresponding 95% CIs for each arm
- Time to progression of bone disease will be calculated using the Kaplan-Meier method. Median time to progression of bone disease will be presented, with corresponding 95% CIs for each arm
- Time to progression of extraskeletal disease will be calculated using the Kaplan-Meier method. Median time to progression of extraskeletal disease will be presented, with corresponding 95% CIs for each arm

#### Clinical benefit endpoints

- Descriptive and graphical summaries will be presented for each of the European Organisation for Research and Treatment of Cancer Quality of Life Group Bone Metastases Module (EORTC QLQ-BM-22) and Brief Pain Inventory (BPI) questionnaires at each time point by arm. The EORTC QLQ-BM-22 and BPI will be summarised by each domain.

### Study organisation and administration

The CARBON study is funded by Bayer Healthcare, supported by Yorkshire Cancer Research (YCR) through the YCR Centre for Early Phase Clinical Trials, and is sponsored by the University of Leeds. Additional support is also provided by the National Institute for Health Research (NIHR) through the use of the Clinical Research Network (CRN). Trial supervision will be established according to the principles of Good Clinical Practice and in line with the relevant Research Governance Framework within the UK and through adherence with CTRU standard operating procedures. A Standard Protocol Items: Recommendations for Interventional Trials (SPIRIT) Checklist has been prepared for this manuscript (Additional file 1).

The trial is registered (ISRCTN92755158 and EudraCT number 2015–003979-29).

## Discussion

CARBON is an important and timely study as, despite improved outcomes following breast cancer, 70% of participants with advanced disease will develop skeletal The median survival time after the development of bone metastases is approximately 2–3 years despite the current treatment options available.

As detailed above, the use of radium-223 as a therapeutic strategy for bone metastases is appealing due to the supportive pre-clinical and clinical evidence of activity in prostate cancer. The development of radium-223 enables the opportunity to evaluate the toxicity and tolerability of combining systemic therapy with a bone-targeted agent.

The results of this phase IB/IIA study will help inform the design of subsequent phase II and -III studies that will facilitate the translation of this combination into potential benefit for this participant population.

## Additional file

Additional file 1: Standard Protocol Items: Recommendations for Interventional Trials (SPIRIT) 2013 Checklist: recommended items to address in a clinical trial protocol and related documents. (DOC 122 kb)

## Data Availability

Not applicable as the manuscript does not contain any data.

## Abbreviations

1CTP: Pyridinoline cross-linked carboxyterminal telopeptide
AE: Adverse Event
B-ALP: Bone alkaline phosphatase
bd: Twice a day
CARBON: A randomised phase IB/IIA study of CApecitabine plus Radium-223 (Xofigo™) in breast cancer patients with BONe metastases
CRN: Clinical Research Network
CT: Computerised tomography
CTX: Serum C-telopeptide
DLT: Dose Limiting Toxicity
ECOG: Eastern Co-operative Oncology Group
FDA: Food and Drug Administration
i.v.: Intravenous
ITT: Intention to treat
MRI: Magnetic resonance imaging
NCI CTCAE: National Cancer Institute Common Terminology Criteria for Adverse Events
NIHR: National Institute for Health Research
NTX: *N*-telopeptide of type-I collagen
P1NP: *N*-terminal propeptide of procollagen type-1
Radium-223: Radium-223 dichloride
REC: Research Ethics Committee
SAE: Serious Adverse Event
SAR: Serious Adverse Reaction
SRC: Safety Review Committee
SRE: Skeletal-related event
SSE: Symptomatic skeletal event
SUSAR: Serious Unexpected Adverse Reaction
uNTX: Urinary marker *N*-telopeptide of type-I collagen
uNTX-1: Urinary *N*-telopeptide of type-1 collagen
YCR: Yorkshire Cancer Research

## References

[1] Kingsley L. A., Fournier P. G.J., Chirgwin J. M., Guise T. A. (2007). Molecular Biology of Bone Metastasis. Molecular Cancer Therapeutics.

[2] Brown J. E., Cook R. J., Major P., Lipton A., Saad F., Smith M., Lee K.-A., Zheng M., Hei Y.-J., Coleman R. E. (2005). Bone Turnover Markers as Predictors of Skeletal Complications in Prostate Cancer, Lung Cancer, and Other Solid Tumors. JNCI Journal of the National Cancer Institute.

[3] Kohno N, Aogi K, Minami H, Nakamura S, Asaga T, Iino Y, Watanabe T, Goessl C, Ohashi Y, Takashima S (2005). Zoledronic acid significantly reduces skeletal complications compared with placebo in Japanese women with bone metastases from breast cancer: a randomized, placebo-controlled trial. J Clin Oncol.

[4] Body JJ, Diel IJ, Bell R, Pecherstorfer M, Lichinitser MR, Lazarev AF, Tripathy D, Bergstrom B (2004). Oral ibandronate improves bone pain and preserves quality of life in patients with skeletal metastases due to breast cancer. Pain.

[5] Hultborn R, Gundersen S, Ryden S, Holmberg E, Carstensen J, Wallgren UB, Killany S, Andreassen L, Carlsson G, Fahl N (1999). Efficacy of pamidronate in breast cancer with bone metastases: a randomized, double-blind placebo-controlled multicenter study. Anticancer Res.

[6] Conte PF, Latreille J, Mauriac L, Calabresi F, Santos R, Campos D, Bonneterre J, Francini G, Ford JM (1996). Delay in progression of bone metastases in breast cancer patients treated with intravenous pamidronate: results from a multinational randomized controlled trial. The Aredia Multinational Cooperative Group. J Clin Oncol.

[7] Lipton A, Cook R, Saad F, Major P, Garnero P, Terpos E, Brown JE, Coleman RE (2008). Normalization of bone markers is associated with improved survival in patients with bone metastases from solid tumors and elevated bone resorption receiving zoledronic acid. Cancer.

[8] Bruland OS, Nilsson S, Fisher DR, Larsen RH (2006). High-linear energy transfer irradiation targeted to skeletal metastases by the alpha-emitter 223Ra: adjuvant or alternative to conventional modalities?. Clin Cancer Res.

[9] Kerr C (2002). (223)Ra targets skeletal metastases and spares normal tissue. Lancet Oncol.

[10] Henriksen G, Fisher DR, Roeske JC, Bruland OS, Larsen RH (2003). Targeting of osseous sites with alpha-emitting 223Ra: comparison with the beta-emitter 89Sr in mice. J Nucl Med.

[11] Lewington VJ (2005). Bone-seeking radionuclides for therapy. J Nucl Med.

[12] Parker C, Nilsson S, Heinrich D, Helle SI, O’Sullivan JM, Fossa SD, Chodacki A, Wiechno P, Logue J, Seke M (2013). Alpha emitter radium-223 and survival in metastatic prostate cancer. N Engl J Med.

[13] Larsen RH, Saxtorph H, Skydsgaard M, Borrebaek J, Jonasdottir TJ, Bruland OS, Klastrup S, Harling R, Ramdahl T (2006). Radiotoxicity of the alpha-emitting bone-seeker 223Ra injected intravenously into mice: histology, clinical chemistry and hematology. In Vivo.

[14] Nilsson S, Larsen RH, Fossa SD, Balteskard L, Borch KW, Westlin JE, Salberg G, Bruland OS (2005). First clinical experience with alpha-emitting radium-223 in the treatment of skeletal metastases. Clin Cancer Res.

[15] Nilsson S, Strang P, Aksnes AK, Franzen L, Olivier P, Pecking A, Staffurth J, Vasanthan S, Andersson C, Bruland OS (2012). A randomized, dose-response, multicenter phase II study of radium-223 chloride for the palliation of painful bone metastases in patients with castration-resistant prostate cancer. Eur J Cancer.

[16] Coleman R, Aksnes AK, Naume B, Garcia C, Jerusalem G, Piccart M, Vobecky N, Thuresson M, Flamen P (2014). A phase IIa, nonrandomized study of radium-223 dichloride in advanced breast cancer patients with bone-dominant disease. Breast Cancer Res Treat.

[17] Henriksen G, Breistol K, Bruland OS, Fodstad O, Larsen RH (2002). Significant antitumor effect from bone-seeking, alpha-particle-emitting (223)Ra demonstrated in an experimental skeletal metastases model. Cancer Res.

[18] Nilsson S, Franzen L, Parker C, Tyrrell C, Blom R, Tennvall J, Lennernas B, Petersson U, Johannessen DC, Sokal M (2007). Bone-targeted radium-223 in symptomatic, hormone-refractory prostate cancer: a randomised, multicentre, placebo-controlled phase II study. Lancet Oncol.

[19] Sartor O, Coleman R, Nilsson S, Heinrich D, Helle SI, O’Sullivan JM, Fossa SD, Chodacki A, Wiechno P, Logue J (2014). Effect of radium-223 dichloride on symptomatic skeletal events in patients with castration-resistant prostate cancer and bone metastases: results from a phase 3, double-blind, randomised trial. Lancet Oncol.

